# Connectome-based predictive modeling of grip strength: a marker of physical frailty

**DOI:** 10.3389/fnins.2025.1697908

**Published:** 2025-12-04

**Authors:** Amin Ghaffari, Majd Abouzaki, Yasmine Romero, Andrew Sun, Aaron Seitz, Jason Langley, Ilana J. Bennett, Xiaoping Hu

**Affiliations:** 1 Department of Bioengineering, University of California, Riverside, Riverside, CA, United States; 2 Department of Cell, Molecular, and Developmental Biology, University of California, Riverside, Riverside, CA, United States; 3 Department of Biochemistry, University of California, Riverside, Riverside, CA, United States; 4 Department of Psychology, Northeastern University, Boston, MA, United States; 5 Department of Psychology, University of California, Riverside, Riverside, CA, United States; 6 Center for Advanced Neuroimaging, University of California, Riverside, Riverside, CA, United States

**Keywords:** connectome, frailty, neuroimaging biomarkers, connectome-based predictive modeling, older adults, caudate nucleus

## Abstract

**Introduction:**

Frailty is characterized by a persistent and progressive decline in functional capacity, leading to increased vulnerability to stressors and a heightened risk of adverse health outcomes, both physically and mentally. Despite frailty’s prevalence in older adults, there is limited research on its neural substrates.

**Methods:**

In this study, we used connectome-based predictive modeling (CPM) to find a linear relationship between task-based connectomes taken from tasks that involved similar handgrip manipulations and a separate measure of physical frailty: the maximum grip strength in older adults.

**Results:**

We observed that the task-based connectomes were able to explain individual differences in grip strength, with the Subcortical and Cerebellum network, particularly the caudate nucleus functional connectivity, being the strongest predictor.

**Discussion:**

These findings demonstrate that task-based functional connectomes can serve as personalized markers for predicting individual behavioral measures, such as handgrip strength, and highlight the role of the caudate nucleus in physical frailty.

## 1 Introduction

Physical frailty, which refers to a decline in physical strength and energy, is prevalent in older adults and has been attributed to impaired cognitive function and adverse health outcomes (González-Vaca et al., 2014; Eyigor et al., 2015). The strength of a contraction on a handgrip, known as isometric handgrip strength, has been used as a marker of physical frailty (Dudzińska-Griszek et al., 2017; Chainani et al., 2016). While handgrip strength can partially be explained by muscle properties (e.g., cross-sectional area and architecture) (Weber et al., 2022; Amara et al., 2003), it may also be influenced by neural adaptations, such as intermuscular and intramuscular coordination. Moreover, the brain likely plays a crucial role in regulating contraction force production and coordination (Enoka, 1988). These neural contributions to handgrip strength are understudied but may serve as novel biomarkers of physical frailty in older adults, potentially at the individual level.

There is a growing use of imaging-derived data from different modalities to predict clinical phenotypes and disease risk (Wang et al., 2025; Ghaffari et al., 2025; Lu et al., 2024; Langley et al., 2024; Schöttner et al., 2025). In this context, handgrip strength has been attributed to resting-state functional connectivity within motor and salience networks. For example, within healthy older adults, Seidler et al. (2015) found that higher functional connectivity of the motor cortex to putamen, insula, and cerebellum was associated with higher handgrip strength. Another study investigated whole-brain functional connectivity (i.e., connectome) and observed that higher handgrip strength was associated with greater functional segregation of the salience ventral attention network in older adults at rest (Chong et al., 2024). This highlights the potential of the connectome as a valuable resource for analyzing the neural mechanisms underlying grip strength in aging.

Because the connectome is unique for each person, akin to a “brain fingerprint” (Finn et al., 2015), it may also serve as a personalized marker that can be used to predict their individual behavioral measures (Liu et al., 2018; Cai et al., 2019), including handgrip strength. Brain fingerprints serve as the foundation of connectome-based predictive modeling (CPM), a data-driven approach that maps individualized functional connectivity patterns to behavioral phenotypes, enabling the prediction of behavior with personalized precision (Shen et al., 2017). Researchers have used CPM with either resting-state or task-based connectomes to explain individual differences in cognitive performance (e.g., intelligence, Finn et al., 2015; mnemonic discrimination, Wahlheim et al., 2022; working memory, Yang et al., 2023) and clinical symptoms (e.g., anxiety levels, Wang et al., 2021; internet addiction symptomatology, Feng et al., 2024; autism symptom severity, Horien et al., 2022; compulsion severity in obsessive-compulsive disorder, Wu et al., 2023; depressed and elevated mood severity in bipolar disorder, Sankar et al., 2023). CPM has also been utilized to predict behavioral measures in older adults (e.g., attentional control, Fountain-Zaragoza et al., 2019; trust propensity, Chen et al., 2024), demonstrating the viability of this method for phenotypic prediction of this cohort. Extending this approach to frailty, Zúñiga et al. (2023) applied CPM to predict self-reported frailty indices in older adults using their resting-state connectomes.

While both resting-state and task-based connectomes could be used in CPM, task-based connectomes may provide a more robust characterization of brain–behavior relationships (Jiang et al., 2020; Finn and Bandettini, 2021; Greene et al., 2018). This is because the brain state is manipulated based on the task being performed (Greene et al., 2018), shifting the brain connectivity pattern into a more task-relevant state that could be more informative for task-related behavioral measures. Given this closer brain–phenotype coupling, it is important to evaluate whether task-based connectomes derived from tasks sharing similar components maintain individual specificity and predictive power. Such validation is critical to ensure the generalizability and extendibility of CPM across different task conditions.

To better understand the neurobiological basis of physical strength in older adults, a CPM-based predictive model can be developed using connectomes from tasks involving motor components. Such tasks shift the brain into a more alert, motor-relevant state, offering greater sensitivity to motor-related conditions like frailty compared to resting-state connectivity. In this study, we focused on healthy older adults and had them perform two perceptual discrimination tasks on two different fMRI sessions, both of which involved a non-dominant handgrip manipulation. We aimed to test the identifiability of the task-based connectomes across the sessions and identify the key functional connections (FCs) that provided this identification power, which we predicted would be derived from the shared handgrip component. We also measured participants’ maximum isometric voluntary contraction (MVIC) of their non-dominant hand as an indicator of frailty and neuromuscular health (Horemans et al., 2003; Fillyaw et al., 1987; Meldrum et al., 2007; Brinkmann et al., 1997), and evaluated the task-based CPM’s ability to predict MVIC in older adults. We identified FCs predictive of MVIC, which may partly explain motor-related impairments in frail older adults. Finding such brain-based biomarkers for grip strength could identify potential target sites for motor rehabilitation programs, enhancing the effectiveness of interventions such as Brain-Computer Interfaces (BCIs) and Functional Electrical Stimulation (FES) aimed at mitigating frailty in older adults (Mrachacz-Kersting et al., 2021; Glanz et al., 1996).

## 2 Materials and methods

### 2.1 Participants

Fifty-five older adults recruited from communities near the University of California, Riverside (UCR) aged between 65 and 87 years (29 females, M_age_ = 68.8 yrs., SD_age_ = 5.8 yrs) participated in this study. Inclusion criteria required participants to be free of MRI contraindications (e.g., non-compliant implants, claustrophobia), have no significant health problems, no history of drug or alcohol abuse, no hearing or uncorrected vision loss, no psychiatric or neurological condition, right-handedness, and not take psychotropic medications. The participants were screened for normal cognition using the telephone version of the Montreal Cognitive Assessment (MoCA). All participants gave informed consent in accordance with the institutional review board at UCR and received financial compensation for their participation.

### 2.2 Isometric handgrip contraction

At the beginning of each testing session, participants were asked to squeeze a digital hand dynamometer (Vernier Software & Technology, Beaverton, OR) at maximum power with their left (non-dominant) hand. They repeated this three times, and the values were averaged to calculate their MVIC of that session. The final MVIC for each participant was the average of their MVIC values across the testing sessions. This was the parameter of interest to be predicted using the functional connectome.

Before starting the first fMRI run in each testing session, the MVIC of each participant was measured again inside the scanner using a fiber optic MRI dynamometer from Current Designs, Philadelphia, PA. The procedure for this measurement was the same as that of the outside-scanner MVIC. The inside-scanner MVIC was recorded to determine the values required for the participants to perform high grip (HG) and low grip (LG) trials during the fMRI runs (see section 2.4). It should be noted that the inside-scanner dynamometer was not linear across the entire range of force, so the outside-scanner one was used to compare MVIC across the participants.

### 2.3 Image acquisition

Following the MVIC measurement in each task session (see section 2.4), participants went into a Siemens 3 T Prisma (Siemens Medical Solutions, Malvern, PA) scanner at the UCR Center for Advanced Neuroimaging and MR images were acquired using a 64-channel receive-only head/neck coil (or a 32-channel receive-only head coil if participants did not fit in the 64-channel head/neck coil). A T_1_-weighted MPRAGE scan [repetition time (TR)/echo time (TE)/inversion time = 2400/2.72/1060 ms, field of view (FOV) = 256 × 240 mm^2^, 208 slices, 0.8 mm^3^ isotropic resolution, GRAPPA = 2] was acquired to be used as the reference for registration of the functional scans to the anatomical space and then the Montreal Neurological Institute (MNI) space. Five runs of a gradient echo-planar imaging (EPI) sequence (TR/TE = 1750/32 ms, FOV = 221 × 190 mm^2^, 72 slices, 1.7 mm^3^ isotropic resolution, GRAPPA = 2, multiband factor = 3, 7.5 min, 245 TRs) measured the Blood Oxygen Level Dependent (BOLD) signal while participants performed either the auditory or visual task. Additionally, two spin-echo EPI scans were collected (TR/TE = 7,700 ms/58 ms, FOV = 221 × 190 mm^2^, 72 slices, 1.7 mm^3^ isotropic resolution) with anterior-to-posterior and posterior-to-anterior phase encoding directions for susceptibility distortion correction.

### 2.4 Task paradigm

**Figure 1:**
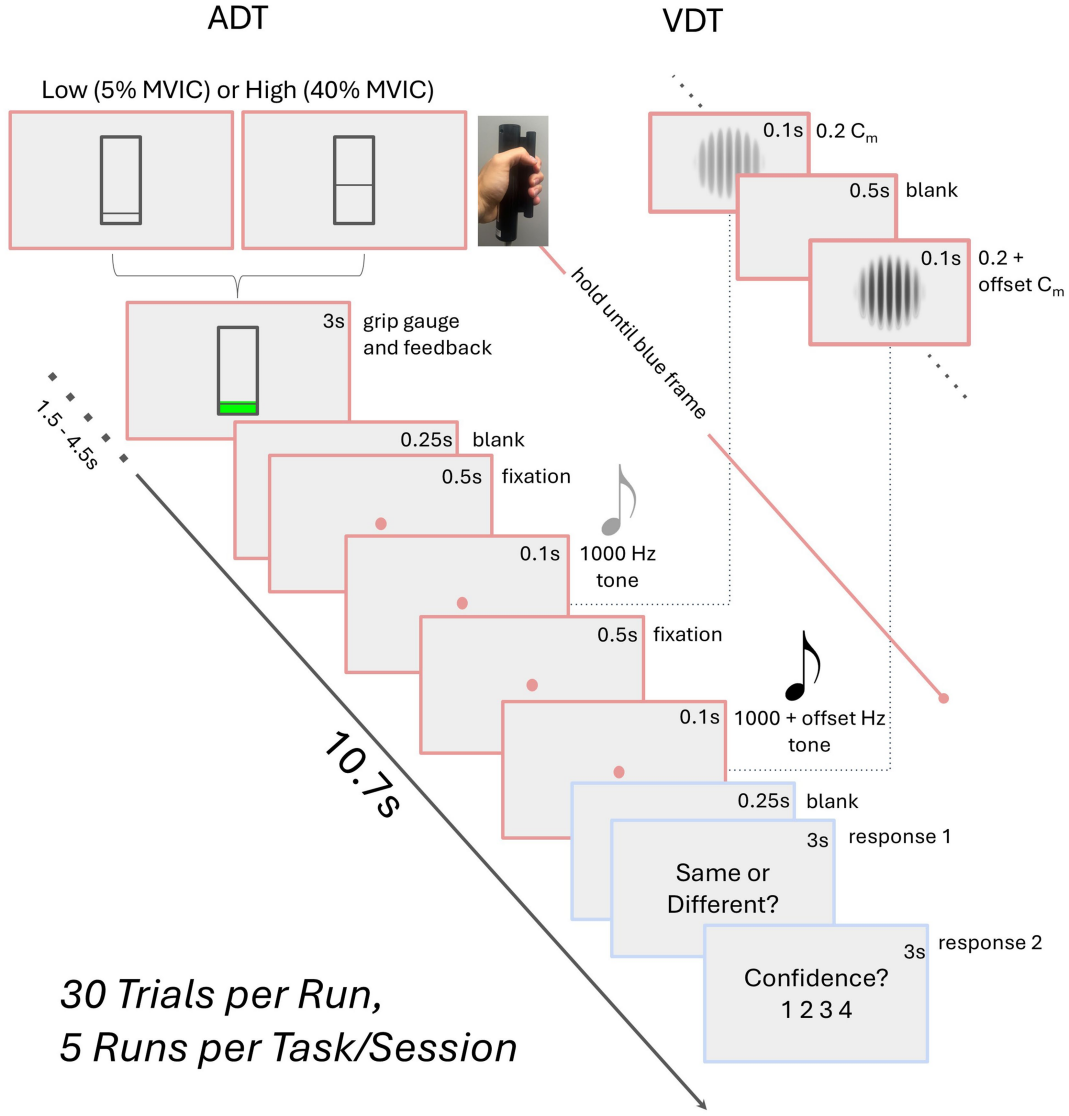
Schematic of the task paradigm. The auditory (ADT) and visual (VDT) tasks share a similar trial structure, differing only in stimuli—two visual Gabors for VDT with certain Michelson contrast (C_m_) and two tones for ADT. Participants perform a high (HG) or low grip (LG) squeeze on a dynamometer, maintaining it until the second stimulus ends. They then relax, indicate whether the stimuli were the same, and rate their confidence. Block durations are provided in seconds.

Participants underwent two fMRI testing sessions on separate days, completing the auditory discrimination (ADT) task in one session and the visual discrimination (VDT) task in the other (see Figure 1), with a counterbalanced order across the participants. Each task session consisted of five fMRI runs that included 30 event-related trials. Each trial started with participants squeezing a dynamometer for 3,000 ms at either 5% (low-grip, LG; 50% of trials) or 40% (high-grip, HG; 50% of trials) of their in-scanner MVIC. This manipulation was intended to modulate arousal (Mayhew et al., 2017; Sterr et al., 2009), but is of interest in the current analysis because it involved a handgrip demand on each trial. The squeeze was followed by a blank screen (250 ms), fixation dot (500 ms), task-specific initial stimulus (100 ms), fixation dot for ADT or blank screen for VDT (500 ms), and task-specific second stimulus (100 ms). At this stage of the trial, participants were asked to relax their grip, and there was a fixation dot (250 ms) on the screen followed by a prompt to respond whether the stimuli were the “same” or “different” (3,000 ms). Finally, another prompt appeared for the participants to indicate their confidence in the prior response (3,000 ms). In the ADT task, the initial stimulus was always a standard tone (1,000 Hz). The second stimulus was either a standard tone or an oddball tone that was offset from the standard by +8, +16, +32, or +64 Hz (6 trials each per run). In the VDT task, the initial stimulus was always a standard visual Gabor patch (6 cycles per degree, 4-degree diameter; Michelson contrast (C_m_) of 0.2 between the maximum and minimum luminance values of the Gabor patch). The second stimulus was either the standard Gabor patch or an oddball Gabor patch that was offset from the standard Gabor’s contrast by 0.06, 0.12, 0.24, or 0.48 Michelson contrast (6 trials each per run). Average accuracy, measured as the percent of correct same/different responses to the second stimulus, was calculated for each contrast level and each task. Confidence ratings were not considered in the current study.

### 2.5 Preprocessing

Raw fMRI and T1-weighted DICOM files were converted to NIFTI files using *dcm2niix* (Li et al., 2016). Then, the T1-weighted structural scans of the participants were skull-stripped using *BET* from FMRIB Software Library (FSL).^1^ The skull-stripped brain was segmented into white matter (WM), gray matter (GM), and the cerebrospinal fluid (CSF) using FSL’s FAST, and the corresponding masks were extracted. The fMRI images were corrected for slice timing, motion, and susceptibility distortions using FSL’s *slicetimer, mcflirt,* and *topup,* respectively. Spatial smoothing was performed using a Gaussian filter with a full width at half maximum of 2 mm.

A transformation was derived from the average functional preprocessed scan of each participant to their T_1_-weighted image using a rigid body transform with a boundary-based registration cost function. Next, a transformation was derived from each participant’s T_1_-weighted image to standard space using a non-linear transformation. The two transformations were concatenated, and each fMRI series was transformed to the MNI space using the resulting transformation. Transformed images were visually examined to ensure accurate registration.

### 2.6 Functional connectomes

**Figure 2:**
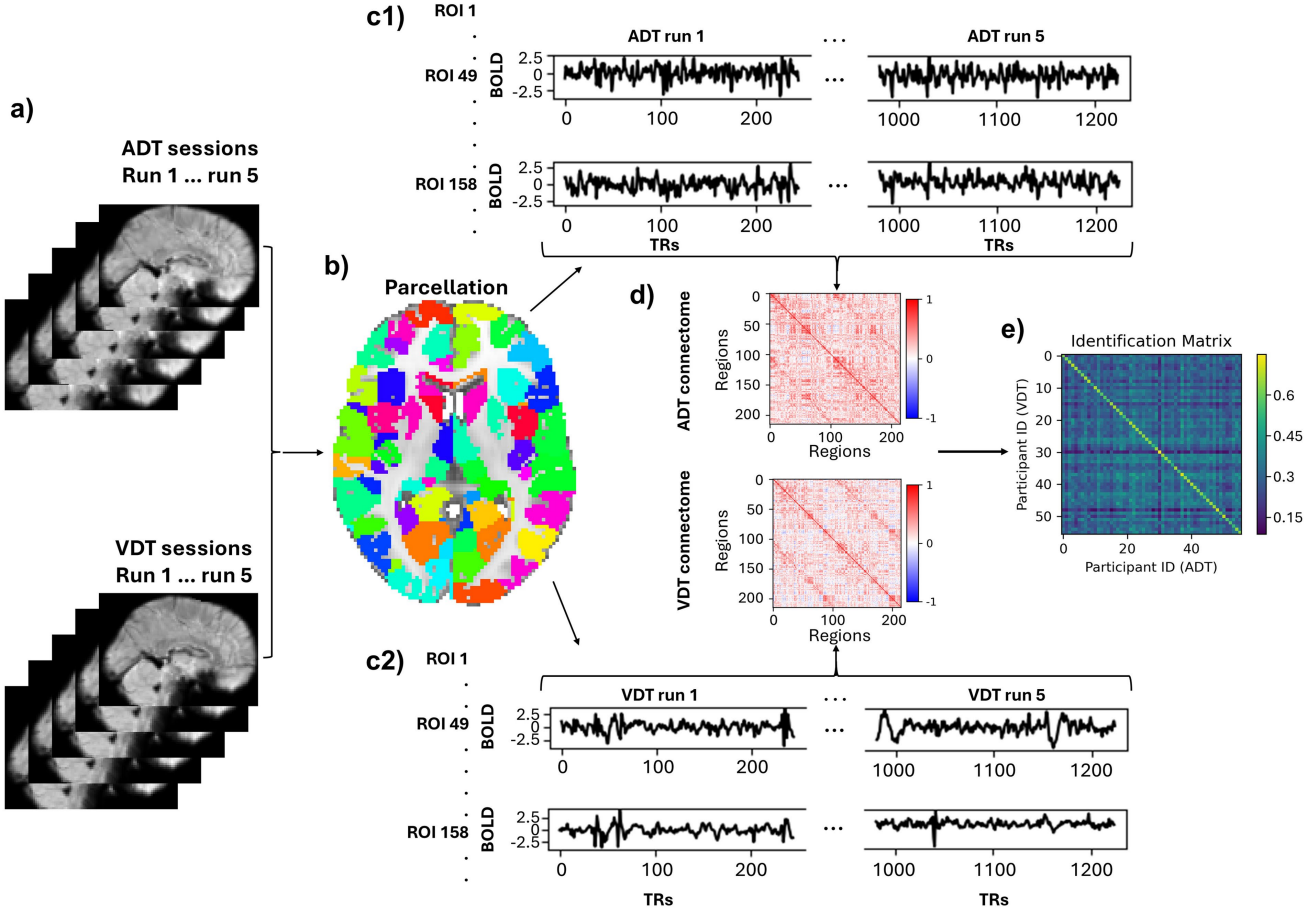
Pipeline of deriving participant-based connectomes and identification. **(a)** A sample fMRI sagittal volume of a subject for each run of each task **(b)** An axial view of the parcellation. **(c1,c2)** Time courses of two sample regions (49, 158) normalized and concatenated across the runs. **(d)** A sample subject’s connectome of the ADT and VDT. **(e)** Identification matrix. The diagonal elements represent the correlation between the same participants’ connectomes across the two task sessions.

Parcellation was performed for each run separately (Figure 2a), where the time series of voxels within each ROI (node) were averaged to extract the time series of that region. We used Shen et al. 268-region atlas (Shen et al., 2013) to define regions of interest (ROIs) in MNI space (Figure 2b). This parcellation’s regions are grouped into eight functional networks: Medial Frontal (MF), Frontoparietal (FP), Default Mode (DM), Subcortical and Cerebellum (SC), Motor (M), Visual I (VI), Visual II (VII), and Visual Association (VA). VI and VII were combined into a single visual network (VIS) because some ROIs in VII were outside the field of view in this study. Additional ROIs, particularly in the cerebellum, were also excluded, resulting in a parcellation of 215 ROIs across seven functional networks.

At this stage, six motion parameters (3 translations and 3 rotations) as well as the average signals of the white matter, cerebrospinal fluid, and global signal were regressed out of each ROI’s time series. Linear trends were also removed from each ROI’s time series and a low-pass filter (0.2 Hz) was applied. Subsequently, the region-specific time series were z-scored within each run, and concatenated across the five runs for each session, which resulted in a time series of 1225 (245×5) time points for each ROI (Figure 2c) in each session. Finally, Pearson’s correlation coefficient was measured between each pair of the ROIs’ time series to produce the participant-specific connectomes for each session (Figure 2d). By only considering the lower triangular part of the connectomes and applying Fisher’s r-to-z transformation to the resulting vector, the functional brain fingerprint of each participant for each session was acquired.

### 2.7 Identification

As described above, each participant’s functional connectome was computed separately for the ADT and VDT sessions. To evaluate the identification accuracy of the connectome “fingerprints,” an identification matrix was constructed by calculating the Pearson correlation coefficient between every participant’s ADT connectome and every participant’s VDT connectome (Figure 2e). In this matrix, each row stores a participant’s VDT fingerprint correlation coefficient with all participants’ ADT fingerprints, and if the maximum of the row is the diagonal element, it indicates a correct ADT identification. The same procedure was used for identifying VDT scans within each column using the connectome from the ADT scans. To confirm that the observed identification accuracy was not due to chance, we conducted additional control analyses. A permutation test with 1,000 random shuffles of participant labels was used to estimate a null distribution of chance-level accuracies. Other control analyses were performed to assess whether confounding factors (session, motion, or total brain volume) contributed to the identification accuracy. We regressed out session order (i.e., whether the auditory or visual task occurred first), motion outliers (number of volumes with framewise displacement (FD) > 0.2 mm), and total brain volume (gray matter + white matter) from the functional connectomes and repeated the identification procedure. We also performed a permutation test by randomly shuffling total brain volume across participants to confirm that identification accuracy was not driven by morphological differences.

Task-based connectomes are made up of FCs between different ROIs, and each of these FCs (i.e., features or edges) contributes differently to the identification power of the connectomes. We used Differential Power (DP) to measure how discriminative (differentiating) each feature was among participants (Finn et al., 2015). To measure DP, we computed the feature product value (FPV) between each pair of the participants across the ADT and VDT session for each feature,


$$\mathrm{FPV}{\left(\mathrm{fc}\right)}_{\mathrm{ij}}=\mathrm{adt}{\left(\mathrm{fc}\right)}_{i}\times \mathrm{vdt}{\left(\mathrm{fc}\right)}_{j}$$

where i and j are labels of participants, fc represents an FC, and adt/vdt are the functional connectomes of the corresponding participants derived from ADT/VDT. If an FC is discriminative between participants, its FPV when calculated for the same person should have a higher value than for different participants. In other words, a differentiating edge across the functional connectomes has the property that,


$$\mathrm{FPV}{\left(\mathrm{fc}\right)}_{\mathrm{ij}}<\mathrm{FPV}{\left(\mathrm{fc}\right)}_{\mathrm{ii}}\;\text{and}\;\mathrm{FPV}{\left(\mathrm{fc}\right)}_{\mathrm{ji}}<\mathrm{FPV}{\left(\mathrm{fc}\right)}_{\mathrm{ii}}$$

Following the same steps described in previous work (Finn et al., 2015), we computed the empirical probability (p_e_) of each feature in maintaining this characteristic in the dataset,


$$p_{e}=\frac{P(\mathrm{FPV}{\left(\mathrm{fc}\right)}_{\mathrm{ij}}<\mathrm{FPV}{\left(\mathrm{fc}\right)}_{\mathrm{ii}})}{2}+\frac{P(\mathrm{FPV}{\left(\mathrm{fc}\right)}_{\mathrm{ij}}<\mathrm{FPV}{\left(\mathrm{fc}\right)}_{\mathrm{ii}})}{2}$$

The probabilities in this equation can be calculated by counting the number of participants for whom this characteristic is held. Finally, DP is calculated by computing the log-likelihood of the empirical probability across the dataset.


$$\mathrm{DP}(\mathrm{fc})=\sum_{i}\{\;(-L(P_{e}))\}$$

A high DP value is associated with a low value of the p_e_ parameter, meaning that the corresponding FC is more discriminative, and therefore, has contributed more to participants’ correct identification.

### 2.8 MVIC prediction

The ability of functional task-based connectomes to predict average MVIC scores was evaluated using 52 participants, as three were excluded due to missing MVIC scores. We used CPM to predict these scores using connectomes of both tasks. In summary, this method consists of one feature selection and one prediction step in a leave-one-out manner (Shen et al., 2017). While the leave-one-out method may produce higher variance compared to k-fold approaches, it is advantageous for small-sized datasets, as it maximizes the training data available in each fold, improving the stability of the predictive models. Moreover, since each data point serves once as an independent test case, this approach provides a thorough assessment of model performance at the individual level, which is especially valuable for individual-specific predictions. To perform the CPM, first, a subject is set as the test set, and the rest are set as the training set. The training set goes into the feature selection step, where the correlation coefficient of each feature of the connectome with MVIC scores across the training set is computed. The feature selection for each training fold was done through 1,000 bootstrapping resampling with replacement, where at each iteration, a fraction of the training set (randomly chosen among 0.8, 0.85, 0.9, and 0.95) was taken and the correlation between each FC of the connectome of that iteration sample and their MVIC scores was calculated. Such resampling across varying subsets of the training data helps guard against the influence of outliers and improves the stability of selected features (Wei et al., 2020), partially mitigating the high-variance concern associated with leave-one-out approaches. If the *p*-value of a certain FC with the iteration sample MVIC scores is lower than a certain feature selection threshold (FST) in 80% of the bootstrapping iterations, that feature is retained for model building to predict the test set MVIC score. The retained features are separated into two sets of positive and negative features based on the sign of their correlation with the MVIC scores. Then, for each participant, the value of all negative and positive retained features are averaged separately so each participant has a negative and positive feature strength in the training set. Finally, using the difference between negative strength and positive strength values, a linear model is built to predict the MVIC scores of the training set (each participant has only one entry into the model, that is, their strength difference). This model is then applied to the test set and the test participant’s MVIC score is predicted using their strength difference. By doing this procedure for all participants, a vector of the predicted MVIC scores is generated. The performance of the CPM in predicting the MVIC scores is assessed by measuring the correlation coefficient between the predicted and observed scores and is reported in terms of correlation coefficient (r), variance explained (R^2^), and root-mean-square error (RMSE).

MVIC scores are known to be affected by age and sex of the participants, where males usually have a higher score compared to females (Sars et al., 2018; Christou, 2011). For this reason, we considered age and sex as covariates and regressed out their effects from the MVIC scores using a linear model. It should be noted that if the data from the test set is used for covariate regression, it might cause inflated performance of the CPM due to data leakage (Rosenblatt et al., 2024). Therefore, in each fold, we derived a model to regress out the effect of covariates from the training set and applied that model on the test participant to remove the covariates’ effect from the test participant’s MVIC scores. To ensure that CPM performance was not dependent on any single FST, we evaluated the model using three *a priori*–selected FSTs: 0.01, 0.005, and 0.001. Testing multiple thresholds allowed us to assess the robustness of the CPM results and confirm that model performance was not driven by an arbitrary choice of FST. To identify the FCs predictive of MVIC and their potential role in physical frailty, we extracted features consistently chosen in the feature selection step across all folds for each task. We then visualized shared features between the two tasks to ensure the shared features were not task-specific or influenced by outliers.

Permutation testing was conducted for MVIC prediction by randomly assigning MVIC values to brain fingerprints. The CPM with covariate regression was applied to each permuted dataset, independently performing covariate removal, feature selection, and prediction in each iteration. By computing the correlation between predicted and observed MVIC values for 1,000 permutations, a chance-level CPM performance was obtained and compared with the CPM performance on real data.

## 3 Results

### 3.1 Behavioral performance

**Figure 3:**
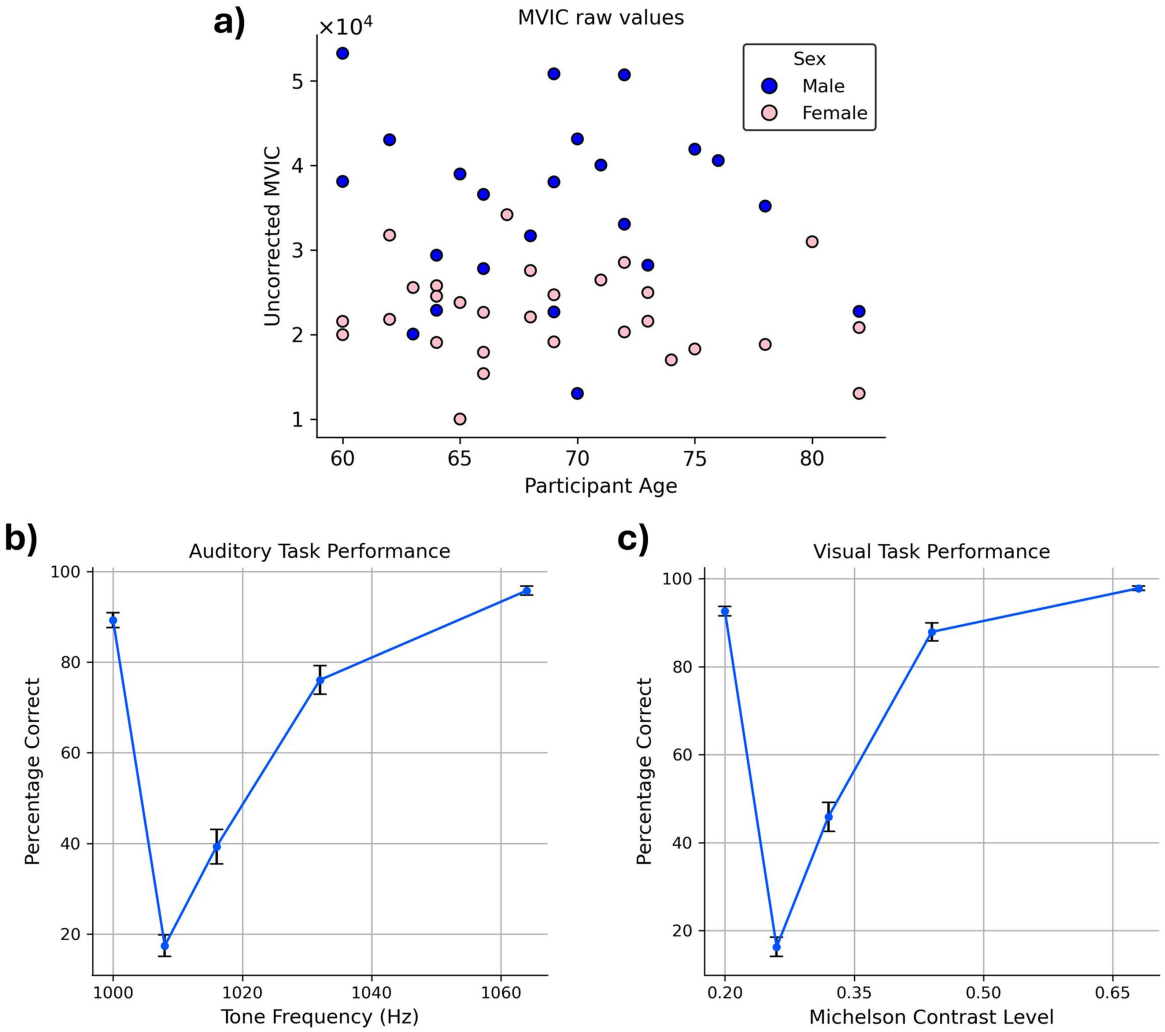
Behavioral task performance. **(a)** MVIC values. The blue and pink circles in the scatter plot correspond to males and females, respectively. MVIC values are reported in an arbitrary unit reported by the dynamometer. **(b,c)** Average accuracy across the participants in **(b)** auditory task **(c)** visual task. Error bars indicate the standard error of the mean (SEM) across participants.

While the partial correlation between chronological age and grip strength—controlling for sex—was not statistically significant in our sample (*r* = −0.10, *p* = 0.46), we regressed out age effects from grip strength to maintain consistency with prior studies (Chandrasekaran et al., 2010; Figure 3a). Regarding sex, between-group t-test revealed that males (34,859.49) had significantly higher average MVIC values than females (22,347.20), *p* < 0.001.

Examination of accuracy data confirmed that participants exhibited high task performance on both the VDT and ADT, as reflected in their response accuracy. Correct “same” responses to standard stimuli were high (VDT: 92.62%; ADT: 89.29%) and correct “different” responses to oddball stimuli increased monotonically as a function of their dissimilarity to the standard stimulus (i.e., as they became easier to distinguish from the standard stimulus; Figures 3b,c).

### 3.2 Identification

For both VDT and ADT, all participants were correctly identified using the fingerprints derived from the other task (identification accuracy = 100%), showing that the task-derived connectomes are highly reliable as individual-specific markers within this cohort. The permutation test confirmed that this result is not likely due to chance (*p* < 0.0001). Identification accuracy remained unchanged after controlling for session order, motion outlier volumes, and structural brain volume, demonstrating that the fingerprinting results were robust to these potential confounds.

**Figure 4:**
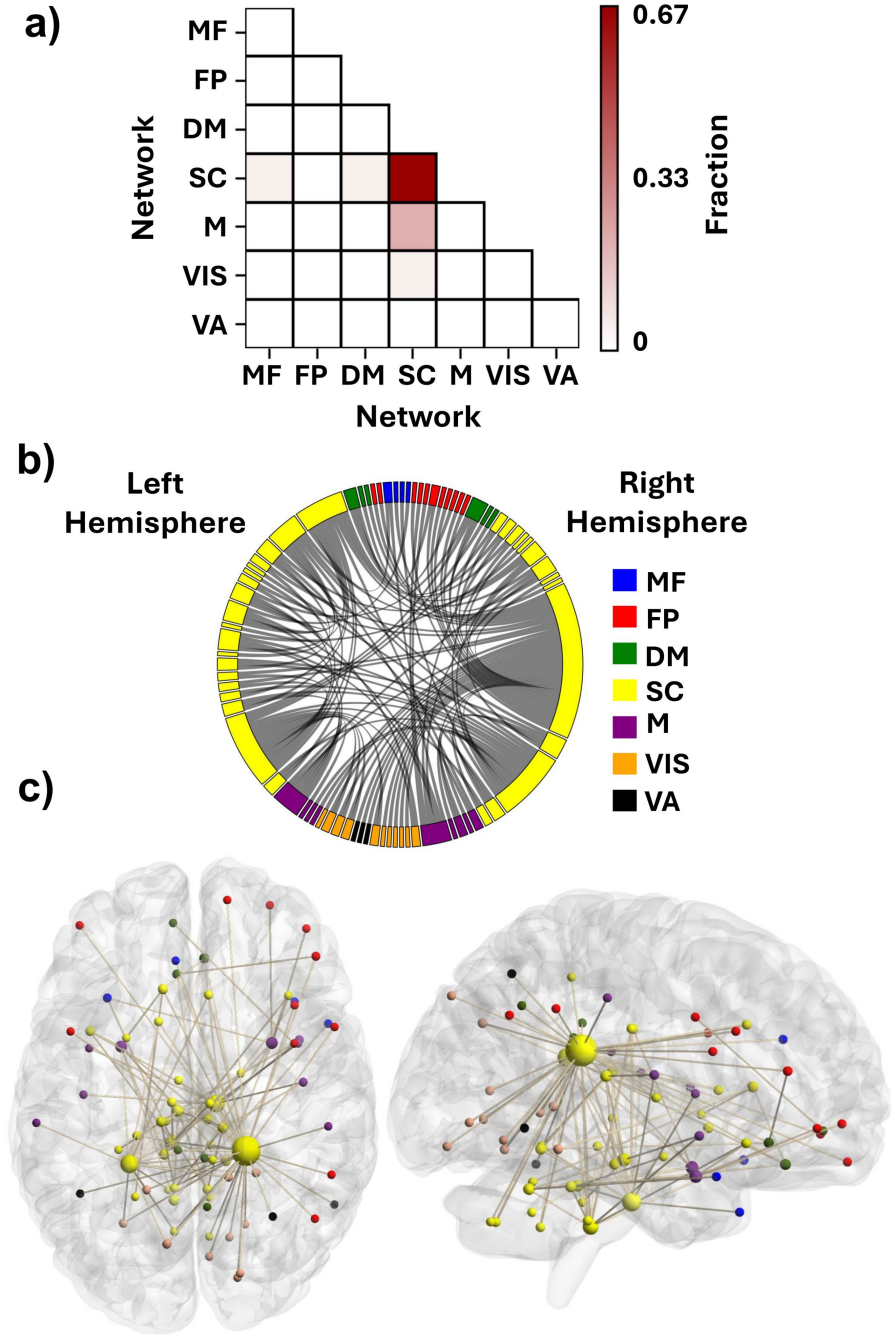
Contributing features to participant identification. **(a)** Fraction of features with suprathreshold differential power within and between the functional networks. Each element shows what fraction of the discriminative edges resides between the networks. **(b)** Distribution of the discriminative FCs among the seven investigated networks. **(c)** An anatomical visualization of the location of the ROIs containing suprathreshold DP features in the brain. Larger regions show a higher number of differentiating FCs. The list of networks is as follows: MF, the medial frontal network; FP, the frontoparietal network; DM, the default mode network; SC, the subcortical-cerebellum network; M, the motor network; VIS, the visual I and II network; VA, the visual association network.

To recognize the FCs with a significant role in the identifiability of the connectomes and their corresponding ROIs, DP of all the FCs was computed and thresholded at 99.5th percentile to visualize the most discriminative edges and their corresponding nodes. The SC network had the most significant role in providing the differentiating edges for the connectome. Approximately 67% of these discriminative features connected the ROIs of the SC network to each other both within each hemisphere and across the hemispheres (Figures 4a,b). The FCs between the SC and M networks also played an important role in identifying the participants, as about 20% of the suprathreshold DP edges connected ROIs of these two networks (Figure 4a), having both inter- and intra-hemispheric edges (Figures 4b,c).

We selected the top three percent of ROIs based on their suprathreshold DP edge count to identify the nodes that contributed most to identification. All of these nodes were categorized within the SC network (Table 1). Interestingly, the top two nodes were contralateral ROIs, left and right caudate tail, which had 38 and 18 high-DP connections, respectively (the two largest yellow spheres in Figure 4c). Most of the right caudate tail’s suprathreshold FCs were to ROIs of the visual system networks (VIS and VA) bilaterally and the right frontoparietal network (Figure 4b). The two caudate regions were followed by ROIs from the brainstem and the cerebellum. Specifically, one ROI encompassed the pons in the right hemisphere of the brainstem; another included both the pons and medulla in the left brainstem, and a third spanned the culmen of the left cerebellar vermis (Table 1), all of which contained both inter- and intra-hemispheric edges.

**Table 1:** Highest contributing ROIs to identification.

Anatomical ROI	# edges	MNI coordinates
Right caudate tail	38	[21.3, −36.4, 22.4]
Left caudate tail	18	[−23.7, −41.3, 19.9]
Right brainstem pons	17	[9.9, −18.8, −30.7]
Left brainstem medulla/pons	12	[−6.9, −33.1, −39.3]
Left vermis culmen	8	[−4.8, −21.5, −15.9]

The ROIs of the SC network with the highest numbers of connections with suprathreshold (99.5 percentile) FCs. The MNI coordinates are the means of the locations of all voxels within each ROI.

### 3.3 MVIC prediction

**Figure 5:**
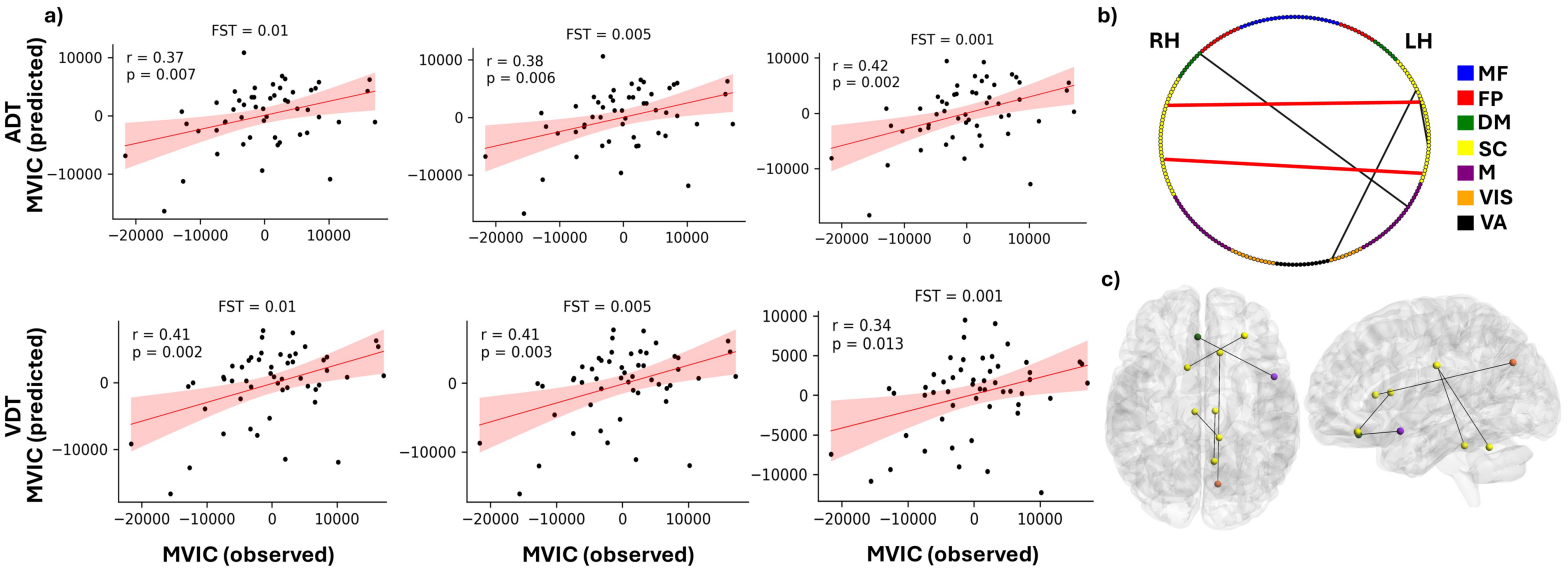
Prediction of MVIC using CPM model. **(a)** Predicted vs. observed MVIC scores after accounting for effects of age and sex for feature selection thresholds of 0.01, 00.5 and 0.001 for both ADT and VDT tasks. “r” shows the correlation between the predicted and observed MVIC scores, and “p” shows the corresponding *p* value of the correlation. **(b)** distribution of the MVIC predictive FCs among the seven investigated networks. RH and LH represent right hemisphere and left hemisphere, respectively. **(c)** An anatomical visualization of the location of the ROIs with the highest number of MVIC predictive features in the brain. The list of networks is as follows: MF, the medial frontal network; FP, the frontoparietal network; DM, the default mode network; SC, the subcortical and cerebellum network; M, the motor network; VIS, the visual network; VA, the visual association network.

For each FST value, the correlation between the predicted and observed MVIC scores was computed. As shown in Figure 5a and Table 2, the CPM model achieved statistically significant prediction accuracy across all thresholds (*p* < 0.05). These results demonstrate that CPM can predict individual differences in MVIC with statistical significance, and this performance is stable and not dependent on the specific FST applied (Table 2). Using ADT connectomes, the best prediction accuracy was obtained at FST = 0.001, yielding a correlation of *r* = 0.42 (R^2^ = 0.103; RMSE = 7420.71, *p*-value = 0.002). For VDT connectomes, with an FST = 0.01, the correlation coefficient between predicted and observed MVIC values was 0.415 (R^2^ = 0.102; RMSE = 7424.29, *p*-value = 0.002).

**Table 2:** CPM performance in predicting MVIC using ADT and VDT connectomes.

FST	ADT				VDT			
	r	*p*-value	R^2^	RMSE	r	*p*-value	R^2^	RMSE
0.001	0.421	0.002	0.103	7420.71	0.342	0.013	0.037	7689.22
0.005	0.376	0.006	0.059	7600.02	0.41	0.003	0.101	7427.388
0.01	0.371	0.007	0.059	7601.65	0.415	0.002	0.102	7424.29

The r column shows the correlation between predicted and observed MVIC scores, the *p*-value indicates its statistical significance, and the R^2^ and RMSE columns report the variance explained and the prediction error, respectively.

Depending on the task and FST, a slightly different set of features passed the bootstrapping threshold and were therefore selected to predict the test set MVIC score for each fold. We extracted the features that were consistently selected across all leave-one-out folds for each task–FST combination (Table 3). Both positive and negative features appeared in different folds of the analysis. However, for FSTs investigated here, no positively correlated features passed the bootstrapping threshold in all folds of the analysis, so the predictive models were predominantly determined by negatively correlated features. In addition, the intersection of the chosen features between the two tasks was examined to ascertain the predictive features that are not specific to either perception task (Table 3). The between-ROI FCs governing the MVIC prediction consistently across the two tasks are shown in Figures 5b,c, along with their corresponding functional networks.

**Table 3:** Number of MVIC-predictive functional connections across tasks and FST thresholds.

FST	Number of selected features		
	ADT	VDT	Intersection
0.001	6	1	0
0.005	12	15	2
0.01	19	36	5

The number of functional connections (FC) correlated with the MVIC scores with a *p*-value less than the feature selection threshold (FST) across bootstrapping iterations are shown for both visual (VDT) and auditory (ADT) tasks. In the intersection column, the number of FCs that had the mentioned property across the tasks are reported.

As shown in Table 3, 19 ADT features and 36 VDT features were consistently predictive of MVIC scores with FST = 0.01 (present in ≥80% of the bootstrapping iterations in each training fold). The overlap between these sets resulted in five FCs that were predictive for both tasks, indicating that these features were not merely driven by the specific task stimuli. Most of these MVIC-predictive features originated from originated from SC network, highlighting the importance of SC connectivity in physical strength. Among these five FCs, there were only two FCs that were also among the predictive features of MVIC across all folds for both tasks when FST was lowered to 0.005 (the FCs shown in red in Figure 5b). As we can see, these two FCs were interhemispheric FCs between ROIs of the SC network. Specifically, one FC was the connection between the right anterior cingulate gyrus and left cerebellum, and the other was the FC between the right caudate nucleus and a region in the left frontal lobe. The right caudate nucleus area was previously shown to contribute strongly to task-based connectome identifiability (Zúñiga et al., 2023). Its predictive role here suggests that this region is not only central to individual identification but also related to physical frailty.

In doing the permutation testing, after shuffling the MVIC scores across the participants, for FST of 0.001, we could not perform the permuted predictions, as in some of the folds, there were no features passing the FST to build the model. Therefore, we performed the permuted predictions using FSTs of 0.005 and 0.01 and found the distribution of correlation between the predicted and observed scores under chance and compared it to the results driven by the unpermuted data. We observed a *p*-value of less than 0.01, indicating that the MVIC prediction was unlikely to have occurred by chance.

## 4 Discussion

With increasing life expectancy, frailty has become a widespread public health concern (Kwak and Thompson, 2021), highlighting the urgent need for predictive models at the individual level to enable early detection and targeted interventions. In this study, we employed CPM, a data-driven approach for phenotypic prediction to explain individual differences in MVIC, an index of physical frailty, among healthy older adults using task-based functional connectomes. We showed that connectomes derived from a task with different perceptual but similar motor components captured individual-specific signatures, enabling CPM to predict MVIC. We identified the key networks (intra-SC, SC-M), ROIs (bilateral caudate tail, pons), and FCs (right caudate tail, right anterior cingulate gyrus) that contributed to the identification of the connectomes and were correlated with MVIC to elucidate connectivity patterns associated with individual differences in grip strength. These findings suggest that CPM may serve as a useful approach for ascertaining predictive markers of frailty; however, further validation is needed before it can be used in clinical applications.

The accuracy for subject identification across the visual and auditory sessions using functional connectomes was 100%, demonstrating the fingerprinting ability of the tasks with shared motor components. Given the modest sample size of our dataset (*N* = 55), high identification accuracy is plausible and has been reported previously in older-adult cohorts with similarly sized resting-state datasets (Ramduny and Kelly, 2025; St-Onge et al., 2023). Moreover, cognitively engaging task paradigms have been shown to further enhance individual identification relative to resting-state scans (Zhang et al., 2022), making our observed level of performance consistent with expectations from prior work. We observed that the FCs within the SC network and between the SC and M networks had the highest contribution to providing identifiability of the task-based connectomes for older adults. This could stem from the handgrip manipulation common to both tasks. Previously it has been shown that engagement in a motor task would alter the motor network connectivity and increase its inter-individual variability (Nettekoven et al., 2015). The motor part of our tasks was a combination of force generation (applying an amount of physical power) and force control (keeping the force in the high grip or low grip range in the squeeze period of the trial) which engages both M and SC networks to coordinate the demands of the task (Spraker et al., 2009). This being the case, the high engagement of these networks would transfer their connectivity patterns to a task-specific state, which makes them highly identifiable. In contrast, studies of resting-state connectomes in younger adults have shown that the majority of discriminative FCs originate from MF and FP networks (Finn et al., 2015; Cai et al., 2021). Older adults tend to have high connectivity in their frontal brain regions which could cause a similar connectivity pattern within MF and FP regions across our cohort of older adults (Goh, 2011), therefore, reducing their corresponding FCs’ differentiation power.

At the ROI level, the bilateral caudate tail areas had the most differentiating FCs across the dataset. The caudate nucleus is one of the subcortical regions known to be correlated with force generation (Sugawara et al., 2023). Task engagement is known to reconfigure functional connectivity of the regions that are directly involved in performance of that task (Cheng et al., 2021; Newton et al., 2007; Harrison et al., 2017). Thus, during the grip portions of the ADT and VDT tasks, caudate functional connectivity pattern would significantly change in the period of force exertion and maintenance (Jiang et al., 2016), which could alter its FC pattern and improve identification accuracy. Because the participants squeezed the dynamometer with their non-dominant hand, it may explain why the right caudate nucleus tail was more involved in the force generation and showed more participant-specific FCs compared to its left counterpart. Following the caudate regions, we identified ROIs in the right brainstem pons and the left brainstem pons extending into the medulla, which exhibited the most differentiating FCs. The basal ganglia-brainstem pathway is key to motor control, helping to integrate voluntary and automatic movements. It regulates postural muscle tone and locomotion by sending signals to brainstem motor networks, which are essential for starting and sustaining movement (Takakusaki et al., 2004). Given this role, its functional connectivity pattern would be altered during a task with a squeeze component, which could contribute to the observed individual differences across the dataset. Furthermore, these brainstem ROIs’ FCs with the right caudate tail were among the features with the highest differential power, showing the contribution of basal ganglia—brainstem connections in individuals’ identifiability. Finally, the culmen area of the vermis of the left cerebellum also had distinct and identifiable FCs. While this area is relatively understudied, its functional connectivity is correlated with gait impairment in Parkinson’s disease patients, which shows its potential significance in motor-related activities (Maiti et al., 2021).

In prediction of MVIC, the ROI corresponding to the right caudate nucleus had an FC correlated with MVIC scores for both tasks. Previous studies showed that brain perfusion in the caudate nucleus is a strong predictor of frailty, distinguishing between frail, prefrail, and nonfrail HIV patients (Luckett et al., 2019), and its volume reduction is associated with frailty in cognitively impaired older adults (Tian et al., 2020). The caudate nucleus and putamen form the neostriatal nucleus, which receives inputs from the cerebral cortex and transmits them to the brainstem and spinal cord, playing a crucial role in the regulation of motor functions (Lee and Muzio, 2020). More importantly, it has been shown that the caudate nucleus, particularly the left side, had the highest number of FCs that were predictive of self-reported frailty index among all brain regions (Zúñiga et al., 2023), highlighting the role of caudate in older adults’ frailty which is supported by our results as well. While we used task-based connectomes and predicted grip strength as an index of frailty, Zúñiga et al. (2023) used resting-state connectomes to predict levels of self-reported frailty. Despite differences in scan methodology, the caudate nucleus was identified as a neural correlate of frailty. This convergence across task-based and resting-state paradigms, and across objective and subjective frailty-related measures, suggests that the caudate nucleus plays a consistent role in the neural processes associated with frailty. The right anterior cingulate gyrus was also shown to possess FC with the cerebellum that is predictive of physical grip strength. While the anterior cingulate gyrus is not directly related to force generation, it plays a vital role in decision making and many other cognitive processes (Apps et al., 2016), which could be involved in performing a task following the prompts given to the participant.

We also observed that at more stringent feature selection thresholds, the features that showed significant correlations with the MVIC scores in all folds of data were negatively correlated with it. Therefore, the predictive network of FCs that predicted MVIC scores was a negative network, while positive networks were unable to do so. This indicates that positive correlations were less stable across folds, while the CPM model robustly captured the consistent negative associations that represented the dominant and most reliable predictive direction. Such an effect has also been observed in other studies that have used CPM to predict behavioral phenotypes such as Autism Diagnostic Observation Schedule for Autism patients (Ma et al., 2023). Another CPM-based study also observed that for prediction of internet addiction symptomatology, only negatively correlated features remained stable across all folds in predicting this score (Feng et al., 2024). Such an effect suggests that, for certain behavioral or physiological traits, the most explanatory features may be negatively correlated, reflecting inhibitory or compensatory neural mechanisms. In this context, stronger functional coupling might indicate increased neural effort or recruitment of additional regions that accompany reduced motor performance. In terms of frailty, it has been seen that increased between-network connectivity of networks such as motor and default mode is associated with worse gait performance in older adults (Droby et al., 2022). Taken together, our results indicate that higher inter-regional coupling may accompany lower motor strength, consistent with the idea that frailty could involve connectivity increases rather than simple loss.

Regarding limitations of the current study, our dataset had a modest sample size (*n* = 52) and relied on internal cross-validation without an external validation set, and the model was developed using healthy older adults. Including a broader range of individuals across the healthy, pre-frail, and clinically frail spectrum and validating the model on independent datasets would demonstrate its generalizability and strengthen its relevance to real-world aging populations. We used grip strength as an indicator of frailty and predicted it with task-based functional connectomes. However, frailty is a multi-faceted condition (Sobhani et al., 2021), and grip strength is only one component of this condition. As such, our findings specifically relate to one physical dimension of frailty, and incorporating additional frailty domains in future work may provide a more comprehensive understanding. Moreover, MVIC is related to various other factors beyond the brain connectome, sex, and age variables that were considered here. These include but are not limited to corresponding muscles’ cross-sectional area (Weber et al., 2022; Amara et al., 2003), fatigue (Taylor and Gandevia, 2008), and sleep deprivation (Skurvydas et al., 2021). Future work that incorporates these factors may result in a more reliable physical strength predictive model. The connectome, which is made of functional connectivity patterns, is an indirect and correlational measure of neural activity, while our results may also be influenced by non-neural factors such as vascular variability (Rangaprakash et al., 2023), physiological noise (Murphy et al., 2013), or global signal fluctuations (Liu et al., 2017). Although motion, global signal, and preprocessing for denoising the fMRI scans were applied, additional physiological regressors (e.g., heart rate, respiration) and vascular measures that can better isolate neural effects from hemodynamic contributions. In terms of whole-brain coverage, several cerebellar ROIs were excluded from the analysis because they were not captured within the FOV for this study. These regions, along with their corresponding FCs with other ROIs, may contain predictive features for frailty, potentially enhancing CPM performance in predicting MVIC. Future research should explore the effects of several factors on MVIC prediction using the functional connectome, such as parcellation scheme, preprocessing and standardization framework (Loaliyan and Ver Steeg, 2024), task paradigm, and the dynamic nature of the brain connectome (i.e., time-varying brain connectivity states) that has been shown to outperform the static connectome in predicting certain behavioral measures (Ghaffari et al., 2025; Bolton et al., 2020).

Despite these limitations, this is the first study to show that a motor task connectome is individual-specific and can predict a frailty index (handgrip strength) in older adults. The SC network contained most of the differentiating and predictive FCs, suggesting its potential involvement in grip strength variability. Notably, the caudate emerged as the most significant region in MVIC prediction, emphasizing the need for further research in its role in frailty.

## 5 Conclusion

In this study, we applied connectome-based predictive modeling on task-based functional connectivity data to predict an index of frailty, handgrip maximum voluntary contraction strength, in older adults. The model demonstrated statistically significant predictive power for individual differences in contraction strength, suggesting that the task-based connectomes can be informative about frailty state for this cohort. The subcortical areas of the brain, specifically the caudate nucleus, played a key role in providing this predictive power for the connectomes. These findings highlight the potential value of subcortical brain regions, particularly the caudate nucleus, as targets for further investigation into the neural basis of frailty and as potential sources for developing targeted interventions or treatments.

## Data Availability

The raw data supporting the conclusions of this article will be made available by the authors, without undue reservation.

## Footnotes

1
www.fmrib.ox.ac.uk/fsl
